# Statistical correlation of nonconservative substitutions of HIV gp41 variable amino acid residues with the R5X4 HIV-1 phenotype

**DOI:** 10.1186/s12985-016-0486-6

**Published:** 2016-02-16

**Authors:** Elena Pacheco-Martínez, Evangelina Figueroa-Medina, Carlos Villarreal, Germinal Cocho, José L. Medina-Franco, Oscar Méndez-Lucio, Leonor Huerta

**Affiliations:** Programa de Maestría y Doctorado en Ciencias Bioquímicas, Universidad Nacional Autónoma de México, Ciudad Universitaria, Distrito Federal, 04510 México; Unidad de Radio Oncología, Instituto Nacional de Ciencias Médicas y Nutrición Salvador Zubirán, Secretaría de Salud, Avenida Vasco de Quiroga No.15, ᅟDistrito Federal, 14080 México; Departmento de Física Teórica, Instituto de Física, Universidad Nacional Autónoma de México, Ciudad Universitaria, ᅟDistrito Federal, 04510 México; Departmento de Sistemas Complejos, Instituto de Física, Universidad Nacional Autónoma de México, Ciudad Universitaria, ᅟDistrito Federal, 04510 México; Centro de Ciencias de la Complejidad, Universidad Nacional Autónoma de México, Ciudad Univesitaria, ᅟDistrito Federal, 04510 México; Departmento de Farmacia, Facultad de Química, Universidad Nacional Autónoma de México, Ciudad Universitaria, ᅟDistrito Federal, 04510 México; Departmento de Immunología, Instituto de Investigaciones Biomédicas, Universidad Nacional Autónoma de México, Ciudad Universitaria, 04510 Distrito Federal, México

**Keywords:** gp41, gp41 hydrophobicity, gp41 loop, gp41 variability, HIV-1, HIV-1 coreceptor, HR2, R5X4 phenotype

## Abstract

**Background:**

The interaction of the envelope glycoprotein of HIV-1 (gp120/gp41) with coreceptor molecules has important implications for specific cellular targeting and pathogenesis. Experimental and theoretical evidences have shown a role for gp41 in coreceptor tropism, although there is no consensus about the positions involved. Here we analyze the association of physicochemical properties of gp41 amino acid residues with viral tropism (X4, R5, and R5X4) using a large set of HIV-1 sequences. Under the assumption that conserved regions define the complex structural features essential for protein function, we focused our search only on amino acids in the gp41 variable regions.

**Methods:**

Gp41 amino acid sequences of 2823 HIV-1 strains from all clades with known coreceptor tropism were retrieved from Los Alamos HIV Database. Consensus sequences were constructed for homologous sequences (those obtained from the same patient and having the same tropism) in order to avoid bias due to sequence overrepresentation, and the variability (entropy) per site was determined. Comparisons of hydropathy index (HI) and charge (Q) of amino acid residues at highly variable positions between coreceptor groups were performed using two non-parametrical tests and Benjamini-Hochberg correction. Pearson’s correlation analysis was performed to determine covariance of HI and Q values.

**Results:**

Calculation of variability per site rendered 58 highly variable amino acid positions. Of these, statistical analysis rendered significantly different HI or Q only for the R5 vs. R5X4 comparison at twelve positions: 535, 602, 619, 636, 640, 641, 658, 662, 667, 723, 756 and 841. The largest differences in particular amino acid frequencies between coreceptor groups were found at 619, 636, 640, 641, 662, 723 and 756. A hydrophobic tendency of residues 619, 640, 641, 723 and 756, along with a hydrophilic/charged tendency at residues 636 and 662 was observed in R5X4 with respect to R5 sequences. HI of position 640 covariated with that of 602, 619, 636, 662, and 756.

**Conclusions:**

Variability and significant correlations of physicochemical properties with viral phenotype suggest that substitutions at residues in the loop (602 and 619), the HR2 (636, 640, 641, 662), and the C-terminal tail (723, 756) of gp41 may contribute to phenotype of R5X4 strains.

**Electronic supplementary material:**

The online version of this article (doi:10.1186/s12985-016-0486-6) contains supplementary material, which is available to authorized users.

## Background

Important features of the HIV-1 induced disease are determined by the interaction of three main classes of viruses with different subsets of CD4+ cells, currently designated as R5, X4 and R5X4 viruses depending on the coreceptor they use to enter cells (CCR5, CXCR4, or both, respectively). CCR5 is expressed mainly by macrophages and the activated/memory T subset, whereas CXCR4 is predominantly expressed by the naïve, but also the memory, subsets of CD4+ T-lymphocytes and by CD4+ T-cell lines. R5 viruses are responsible for transmission and persist through the whole course of the disease in most of patients. The appearance of R5X4 and X4 viruses in blood associates with the onset of AIDS [1].

Entry of the HIV-1 genome into target cells depends on trimmeric complexes of the viral envelope glycoprotein (Env) heterodimer, which is composed of a hypervariable surface subunit (gp120), and a more conserved, though highly variable, transmembrane subunit (gp41) [2]. CD4 binding to gp120 induces the exposure/formation of the binding site for the coreceptor [3]. The gp120-CD4-coreceptor interaction then allows the extension of gp41 and the insertion of the fusion peptide into the target membrane. Current models indicate that packing of three gp41 C-terminal helices into the grooves of a coiled coil formed by the N-terminal helices forms a structure known as the six-helix bundle, enforcing virus-cell membrane fusion [4, 5].

Determinants of HIV-1 coreceptor tropism have been identified mainly in the hypervariable gp120 V3 loop, where a high positive net charge associates with X4 tropism [6, 7]. V1, V2 and V5 loops modulates the V3 effects [8–12]. In addition, experimental evidence of the participation of gp41 in coreceptor recognition has been provided [13–16]. Gp41 contains approximately 346 amino acids and is composed of an ectodomain, a membrane spanning domain, and a long C-terminal tail (CTT). The ectodomain is organized in an N-terminal fusion peptide, two helical regions known as HR1 and HR2, a central loop, and the membrane proximal external region (MPER). In the ectodomain, HR2 concentrates the highest variation rate [17], whereas the C-terminal tail display the higher average diversity in the protein [2]. Theoretical studies have shown the statistical association of gp41 with coreceptor tropism although there is no a consensus about the putative sites implicated [18–21], and congruency with experimental investigations of coreceptor associated mutations [13, 14] is not clear. Given the high variability and adaptive nature of gp41, discordances may be caused by differences in the databases used, as well as to distinct analytical approaches. Thus, while it seems clear that different gp41 domains participate in determination of virus phenotype, the specific changes involved may develop in a complex, context-dependent manner, similarly to the different mutational pathways observed in studies of the correlates of the gp120 sequence with coreceptor tropism [10] or that obtained for resistance to maraviroc of R5-tropic viruses [22].

Unlike other studies, we focused our analysis on the relationship of the hydropathy index and charge of amino acid positions between coreceptor groups in order to determine if general physicochemical properties of gp41 residues correlate with different virus phenotypes. In addition, we focused on highly variable amino acid positions of gp41 since conserved positions are most probably engaged in maintaining the highly stringent structural properties required for membrane fusion. With this purpose, we retrieved amino acid sequences of a set of 2823 HIV-1 strains from all clades with known coreceptor tropism from Los Alamos HIV Database. Consensus sequences were constructed for homologous sequences (those obtained from the same patient and having the same tropism) in order to avoid bias due to sequence overrepresentation. Then, the variability (entropy) per site was determined and amino acid positions with high variability scores or with large differences in variability between coreceptor groups were selected. Next, we performed a statistical analysis for the association of the viral tropism (X4, R5 and R5X4) with the hydropathy index (HI) and charge (Q) of amino acid residues at those positions. Twelve positions were found linked to coreceptor usage in this analysis. We suggest that some of the most gp41 variable residues are involved in the coreceptor recognition process.

## Results

### Variability of gp41

The statistical association between coreceptor tropism and hydropathy index (HI) or charge (Q) of variable amino acids was analyzed for 2823 gp41 sequences from individual viruses with known coreceptor tropism included in Los Alamos Database at January 2014, considering all clades. After alignment and construction of consensus for homologous sequences, a final number of 773 sequences was obtained as follows: 621 R5, 73 X4, and 79 R5X4. Table 1 presents the percentage of consensus sequences of strains with a given coreceptor tropism in genetic subtypes.

**Table 1:** Percentage of R5, X4 and R5X4 strains in different genetic subtypes

Subtype^a^	R5 (621)	X4 (73)	R5X4 (79)	Total
A (37)	78.4	8.1	13.5	100
B (270)	81.5	9.6	8.9	100
C (164)	87.2	7.9	4.9	100
D (52)	61.5	11.5	26.9	100
others (250)	78.8	10	11.2	100

^a^The number of sequences in each subtype and each tropism group is indicated in parenthesis

**Fig. 1:**
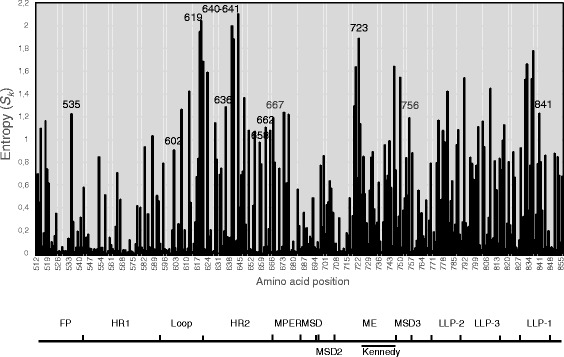
Entropy at each amino acid position (*S*_*k*_) of HIV-1 gp41 calculated from the whole set of sequences. Residue numbers correspond to positions in gp160 HXB2 strain. Residues statistically related to virus phenotype (Tables 2 and 3) are indicated with position number. FP, fusion peptide; HR1, heptad repeat region 1; HR2, heptad repeat region 2; MPER, membrane proximal external region; MSD, membrane spanning domain; MSD2, membrane spanning domain 2; MSD3, membrane spanning domain 3; ME, minor ectodomain; LLP-1, lentiviral lytic peptide 1; LLP-2, lentiviral lytic peptide 2; LLP-3, lentiviral lytic peptide 3

The protein variability calculated by means of the entropy (*S*_*k*_) per site for the whole gp41 sequence is presented in Fig. 1. The highest entropy peaks concentrated at the ectodomain, particularly at positions 619–621 of the C-terminal end of the loop, and 640, 641, and 644 in HR2. In the C-terminal tail, regions with high variability were observed in the putative minor ectodomain (ME) [23–25] and membrane spanning domain three (MSD3) [25, 26], as well as in the lentivirus lytic peptide one (LLP-1). Similar patterns of gp41 variability have been reported before [2, 17].

We considered as highly variable those positions with the highest entropy scores (*S*_*k*_ > 0.9). This criterion yielded 27 positions in the ectodomain and 31 in the transmembrane domain and cytoplasmic tail. Thus, 58 variable positions were considered for statistical analysis of correlation with coreceptor usage (Fig. 1 and Additional file 1: Table S1).

### Relationship of coreceptor usage with hydropathy index and charge of highly variable amino acids

We tested the independence of HI distributions (Mann–Whitney *U* test) and the association of the hydrophobic (HI > 0) or hydrophilic (HI < 0) character (*χ*^2^ test) with coreceptor usage in the R5 vs. X4, R5 vs. R5X4, and X4 vs. R5X4 comparisons. In order to correct for multiple tests we employed the Benjamini-Hochberg procedure by considering false discovery rates (Q_FD_) of 0.05 and 0.1. With both criteria significant *p* values were obtained only for the R5 vs. R5X4 comparison. Additional file 1: Table S1 contains the average and standard deviation of HI at each position in the R5, X4, and R5X4 groups, as well as the *p* values obtained for comparisons between them before correction for multiple tests. Table 2 shows the summary of statistics of positions with significant *p* values after Benjamini-Hochberg correction. Using a Q_FD_ of 0.05, the test of HI-independence distribution (Mann–Whitney *U* test) rendered ten significant amino acid positions. Three of these positions (619, 641 and 667) as well as 602 also showed statistical linkage of hydrophilicity or hydrophobicity (*χ*2) with coreceptor tropism. The same tests were applied to the analysis of correlation of Q with coreceptor usage. Additional file 2: Table S2 shows *p* values obtained for all comparisons before correction for multiple tests and Table 3 contains the summary of significant position statistics after multiple test correction. Statistical independence of Q distribution was found only at position 636, whereas significant association of charged or uncharged character with viral tropism was obtained for this position and for 602 and 658. In total, twelve different positions rendered significant *p* values for HI or Q.

**Table 2:** Summary of statistics of positions significant in the comparison between the hydropathy index of R4X4 and R5 sequences

Mann–Whitney *U* test						
Rank	HXB2 residue	Position^a^	*p* (*U*-test)^b^	*p* (Q_FD_ = .05)^c^	*p* (Q_FD_ = .1)^c^	Location
1	L	619	**.00023**	.00086	.0017	Loop-HR2
2	A	667	**.00033**	.0017	.0034	MPER
3	S	640	**.00064**	.0026	.0052	HR2
4	R	841	**.0009**	.0034	.0068	LLP-1
5	N	636	**.0027**	.0043	.0086	HR2
6	L	641	**.0030**	.0052	.0104	HR2
7	I	756	**.0040**	.0060	.0120	MSD3
8	M	535	**.0061**	.0069	.0138	Fusion peptide
9	E	662	**.0067**	.0078	.0156	MPER
10	T	723	**.0075**	.0086	.0172	Minor ectodomain
11	I	746	.0135	.0095	.0190	Minor ectodomain-MSD3
12	Q	658	.0164	.0103	.0206	HR2
*χ*2 test						
Rank	HXB2 residue	Position	*p* (*X* ^2^)^b^	*p* (Q_FD_ = .05)^c^	*p* (Q_FD_ = .10)^c^	
1	A	667	**.00002**	.00086	.0017	MPER
2	L	602	**.00013**	.0017	.0034	Loop
3	L	641	**.00019**	.0026	.0052	HR2
4	L	619	**.00022**	.0034	.0068	Loop-HR2
5	E	662	.0076	.0043	.0086	MPER
6	V	778	.0080	.0052	.0104	LLP-2

^a^Residue number is based on the sequence of HXB2 gp120

^b^Bold characters indicate positions with significant *p* values using Q_FD_ = .05. Normal characters indicate aditional positions with significant *p* values using Q_FD_ = .1

^c^Benjamini-Hochberg critical *p* values

**Table 3:** Summary of results of statistical comparison between the charge of amino acid positions from R4X4 and R5 sequences

Mann–Whitney *U* test						
Rank	HXB2 residue	Position^a^	*p* (*U*-test)^b^	*p* (Q_FD_ = .05)^c^	*p* (Q_FD_ = .10)^c^	Location
1	N	636	**.0002**	.00086	.0017	HR2
*χ*2 test						
Rank	HXB2 residue	Position	*p* (*X* ^2^)^b^	*p* (Q_FD_ = .05)^c^	*p* (Q_FD_ = .10)^c^	
1	N	636	**.00004**	.00086	.0017	HR2
2	L	602	**.00005**	.0017	.0034	Loop
3	Q	658	**.0010**	.0026	.0052	HR2
4	N	809	.0050	.0034	.0068	LLP-3
5	A	667	.0055	.0043	.0086	MPER
6	R	841	.0078	.0052	.0104	LLP-1

^a^Residue number is based on the sequence of HXB2 gp120

^b^Bold characters indicate positions with significant *p* values using Q_FD_ = .05. Normal characters indicate positions with significant *p* values using Q_FD_ = .1

^c^Benjamini-Hochberg critical *p* values

**Fig. 2:**
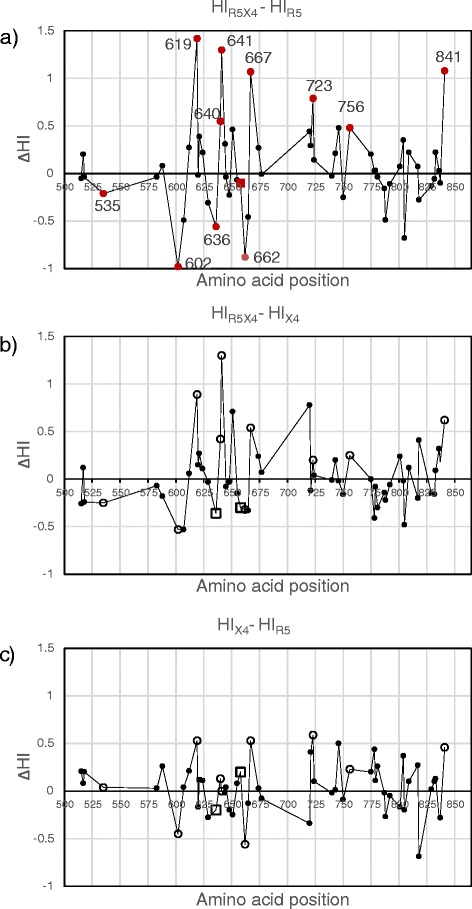
Mean hydropathy index differences (∆HI) of all 58 variable positions included in Additional file 1: Table S1. (a) ∆HI between R5X5 and R5 sequences. Positions showing significant differences of HI (Q_FD_ = 0.05) between R5X4 and R5 viruses are indicated with red circles. Position 658, which exhibited difference in charge only (Table 3) is indicated with a red square. Positions with the largest differences in amino acid frequencies between coreceptor groups (see Fig. 3) are indicated with position number. (b) ∆HI between R5X5 and X4 sequences. (c) ∆HI between X4 and R5 sequences. Positive or negative differences in HI imply a hydrophobic or hydrophilic tendency, respectively, for R5X4 (a, b) or X4 (c) sequences. Note that positions that were significant in the R5X4-R5 comparison (a) where not significant for the comparisons shown in (b) and (c), and are presented to illustrate the diminution of the hydrophobic or hydrophilic tendency of the respective residues (white circles and squares)

Figure 2 compares the mean hydropathy value of all 58 variable residues (listed in Additional file 1: Table S1) among coreceptor groups. Red markers indicate positions that produced significant *p* values with a Q_FD_ of 0.05 showed in Tables 2 and 3. According with statistical analyses, the largest differences in HI were observed for the R5X4-R5 comparison (Fig. 2a). Large increments of hydrophobicity in R5X4 respective to R5 sequences were observed at positions 619, 641, 667 and 841, and moderated increments at 640, 723 and 756, whereas increased hydrophilicity in R5X4 respective to R5 sequences was observed at positions 602, 636 and 662. Position 658, which showed significantly different Q between R5X4 and R5 sequences, is indicated with a red square. A similar pattern, although not significant, was observed in R5X4 respective to X4 sequences (Fig. 2b) and only minor differences were observed in X4 with respect to R5 sequences (Fig. 2c).

**Fig. 3:**
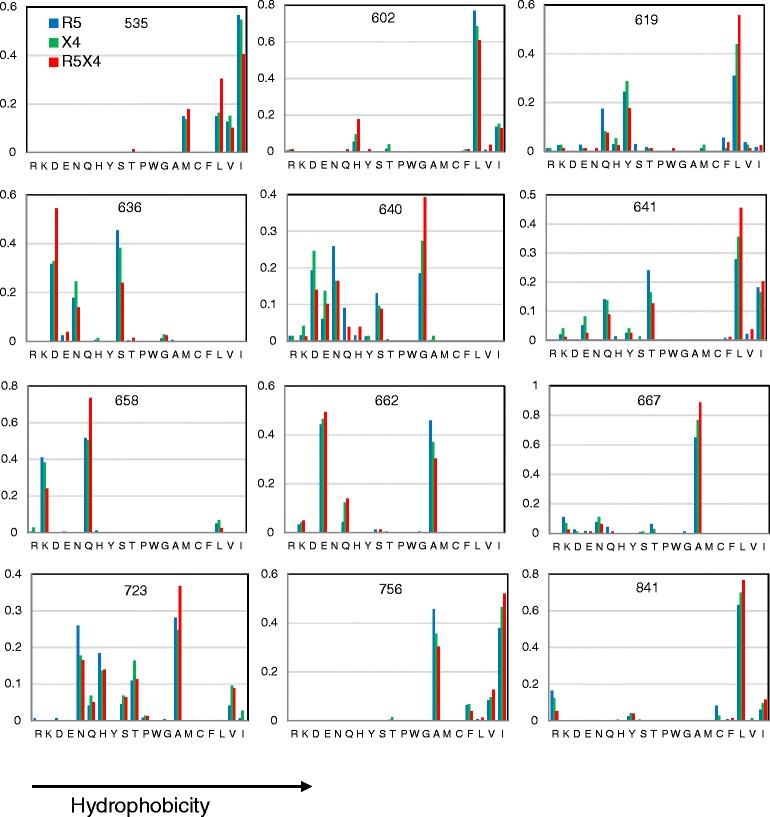
Amino acid distribution at positions statistically related to virus phenotype in the R5, X4 and R5X4 groups

Figure 3 shows a survey of the frequency distribution of particular amino acids at these sites. The major differences between coreceptor groups were at positions 619, 636, 640, and 641. The content of hydrophobic residues at positions 619, 640 and 641 was between 38 and 52 % greater in R5X4 than in R5 sequences, whereas the content of charged residues at position 636 was 40 % greater in R5X4 sequences. Positions 535, 602, 658, 662, 667, 723, 756 and 841 exhibited differences between 18 and 34 % in the content of particular residues.

In summary, taking into account the extent of differences in hydropathy and charge, as well as the frequency distribution of amino acids, a tendency to a hydrophobic character at positions 619, 640, 641, 723 and 756, and to charged amino acids at position 636 and 662, were found in R5X4 respective to R5 sequences.

In order to detect differences in HI or Q in other comparisons (R5X4 vs. X4 and R5 vs. X4), a statistical evaluation was performed by broadening the criterion of false discovery rate. Considering a value of Q_FD_ = .10, again the R5 vs. R5X4 comparison was the only that provided statistically relevant sites. In addition to positions obtained using a Q_FD_ = .05, differences in HI were obtained at positions 746 and 778, whereas different charge was observed at 809 (Tables 2 and 3).

### Correlation between sites

A covariation analysis was performed for positions that were statistically different between coreceptor groups in order to assess if HI or Q values change in a correlated manner. Given the highly organized structure of gp41, it is predictable that many positions should covariate significantly, which is necessary to maintain the structure and function of the protein. However, a higher correlation index for a pair of residues in one tropism group respective to others would be indicative of a complementary contribution to virus phenotype. Thus, the analysis was performed separately on the R5, X4 and R5X4 groups. The covariance analysis also provides information about the positive or negative correlation between values, providing an assessment, for example, of the tendency to hydrophobicity of a pair of residues (positive correlation), or a tendency to hydrophobicity of one residue along with a tendency to hydrophilicity of another (negative correlation).

Table 4 contains Pearson’s correlation coefficients (*r*) for hydrophaty index of pairs of positions in the R5, X4, and R5X4 groups. As expected, most of residue pairs covariate significantly with moderate or high correlation coefficients. However, pairs 602–640, 602–723, 619–640, 636–640, 640–662, and 640–756 correlated with higher *r*’s (>0.4) in the R5X4 group than in the R5 and X4 groups (indicated with bold characters in the column R5X4 in Table 4). Of these, a positive correlation was observed for the 619–640 and 640–756 positions, in agreement with a hydrophobic tendency observed for these residues in R5X4 sequences (Fig. 2a). Instead, negative correlations were observed for the 602–640, 602–723, 636–640 and 640–662 pairs in the R5X4 group, accordingly with the opposite hydrophaty tendencies of these residues in this group observed before (Fig. 2a). Noticeably, position 640 participated in five of six of these covariations, emphasizing the importance of the hydrophobic character of the 640 residue for the R5X4 phenotype.

**Table 4:** Pearson’s correlation coefficients (*r*) for hydrophaty index in gp41 alignments of R5, X4, and R5X4 sequences^a^

		R5		X4		R5X4	
Position 1	Position 2	***r***	*p*	***r***	*p*	***r***	*p*
535	619	−0.18	5.7E-06	−0.38	5.5E-04	−0.31	4.3E-03
535	636	0.26	5.7E-11	0.32	3.2E-03	0.29	6.9E-03
535	641	−0.23	8.1E-09	−0.34	1.8E-03	−0.18	5.3E-02
535	662	0.15	7.7E-05	0.36	7.9E-04	0.11	1.8E-01
535	723	−0.29	2.7E-13	**−0.47**	2.0E-05	−0.27	9.6E-03
535	756	−0.26	8.8E-11	−0.39	2.7E-04	−0.25	1.6E-02
602	619	−0.20	3.3E-07	−0.16	8.3E-02	−0.14	9.4E-02
602	640	−0.22	2.4E-08	−0.15	1.1E-01	**−0.44**	5.9E-05
602	641	−0.17	7.4E-06	−0.11	1.7E-01	−0.16	8.1E-02
602	662	0.20	9.6E-07	0.20	4.5E-02	0.22	2.1E-02
602	723	−0.22	3.3E-08	−0.10	2.1E-01	**−0.40**	1.8E-04
602	756	−0.18	4.2E-06	−0.25	1.5E-02	−0.34	1.2E-03
619	636	−0.28	4.3E-12	−0.38	3.6E-04	−0.28	7.6E-03
619	640	0.20	2.6E-07	0.17	7.9E-02	**0.47**	2.6E-05
619	641	0.20	2.2E-07	0.24	2.0E-02	0.10	1.9E-01
619	658	0.16	2.9E-05	0.21	3.6E-02	0.16	8.2E-02
619	662	−0.45	2.0E-29	−0.51	1.4E-05	−0.54	6.4E-07
619	723	0.45	6.3E-29	0.44	1.1E-04	0.45	3.8E-05
619	756	0.41	2.2E-24	0.39	4.5E-04	0.45	2.1E-05
619	841	0.25	2.5E-10	−0.03	3.8E-01	0.36	6.4E-04
636	640	−0.24	4.0E-09	−0.20	3.8E-02	**−0.58**	2.3E-07
636	641	−0.35	6.7E-18	−0.19	5.1E-02	−0.30	3.6E-03
636	662	0.19	1.2E-06	0.28	1.0E-02	0.21	2.8E-02
636	723	**−0.53**	1.7E-37	**−0.42**	2.0E-04	−0.28	7.6E-03
636	756	**−0.48**	1.4E-35	−0.31	4.0E-03	−0.23	2.3E-02
640	641	0.28	1.7E-13	0.35	1.5E-03	0.35	1.2E-03
640	662	−0.21	4.3E-08	−0.31	3.9E-03	**−0.48**	1.4E-05
640	723	0.35	3.3E-18	0.35	1.1E-03	0.39	3.5E-04
640	756	0.29	1.5E-12	0.16	8.8E-02	**0.45**	1.9E-05
641	723	**0.40**	1.8E-23	**0.52**	7.0E-06	0.23	2.6E-02
641	756	0.36	6.4E-19	0.20	3.9E-02	0.16	7.3E-02
658	662	−0.18	2.5E-06	−0.26	1.1E-02	−0.13	1.2E-01
658	723	0.26	3.2E-10	0.09	2.5E-01	−0.16	7.2E-02
658	756	0.21	3.4E-07	0.08	2.4E-01	0.16	7.2E-02
662	723	−0.45	1.5E-29	−0.42	1.5E-04	−0.49	6.9E-06
662	756	−0.40	4.6E-24	−0.45	6.0E-05	−0.44	2.7E-05
662	841	−0.36	1.5E-19	−0.12	1.3E-01	−0.23	2.2E-02
723	756	0.71	1.5E-66	0.61	1.3E-07	0.67	1.5E-09
723	841	0.20	3.0E-07	0.03	4.3E-01	0.10	2.0E-01
756	841	0.24	1.5E-09	0.07	2.8E-01	0.19	4.9E-02

^a^ Only correlations with a *p*-value less than .0001 for at least one coreceptor group are shown

Correlation with *r* > 0.4 was also observed for the pairs 636–723 and 641–723 in both R5 and X4 groups (indicated with bold characters in the R5 and X4 columns in Table 4), but not in the R5X4 group, indicating that R5 and X4 sequences share hydropathy features at these positions.

Regarding charge, no correlations with *r* > 0.4 between positions were observed (Additional file 3: Table S3).

## Discussion

**Fig. 4:**
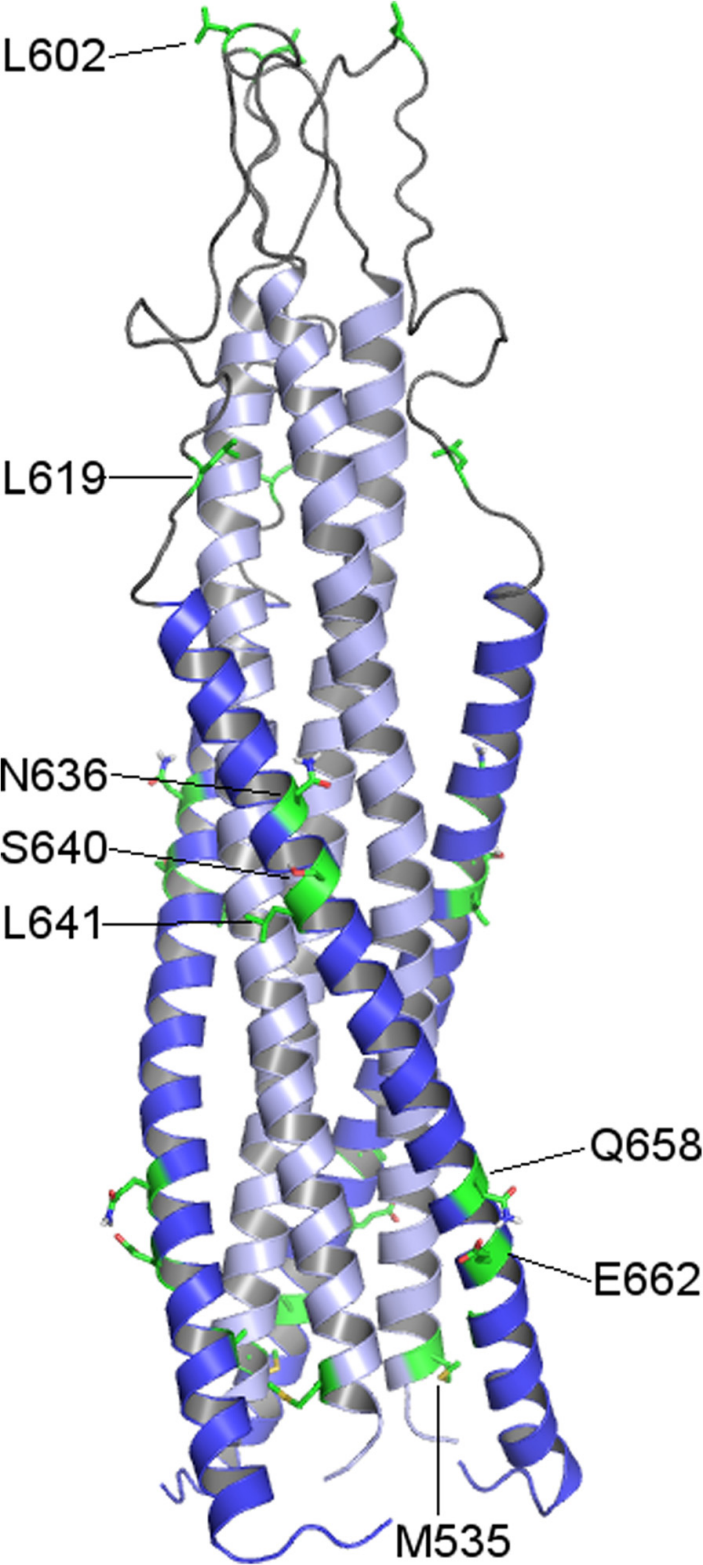
Three-dimensional representation of the trimeric gp41 protein ectodomain. Ribbon representation of the protein with the HR1 domain (positions 531–591) in light blue and the HR2 (positions 624–681) domain in blue. Positions relevant for the R5 or R5X4 tropism (see Fig. 2) are shown in green. The image was obtained from a consensus homology model generated with Prime software [49] from gp160 (Uniprot: Q70626, positions: 531–681) of HIV-1 group M subtype B (isolate LW123), and using two templates (PDB ID's: 2X7R and 1IF3) [47, 48]. The coordinates of this structure are available in the Additional file 4: Figure S1 (Gp41 coordinates - Homology model)

Our results indicate that the R5X4 phenotype associates with a hydrophobic tendency of positions at the C-terminal half of the loop (619) the HR2 (640, 641), the so called minor ectodomain (723), and the putative MSD3 (756), as well as with a hydrophilic/charged tendency in a residue at the disulfide bridge region of the loop (602), and the HR2 (636, 662). The location of the nine positions belonging to the ectodomain is shown in the structure of the six-helix bundle in Fig. 4. Since this study is correlative, it does not necessarily implicates that these residues establish contact with coreceptor molecules, but only that hydrophobic or hydrophilic residues at these positions are more frequently harbored by R5X4 than R5 and X4 viruses. However, it can be speculated that they may contribute to virus phenotype by several mechanisms. Position 602 is the most variable site in the disulfide bridge region of the loop (Figs. 1 and 4). It is known that hydrophobicity of the loop is important for the stability of the gp120-gp41 association [27], so a hydrophilic residue at position 602 may favor gp120 shedding and fusion. Position 619 is part of the LEQ – leucine-glutamate-glutamine in the HXB2 strain – highly variable triplet located at the loop-HR2 boundary (Fig. 4). To our knowledge, there are no experimental studies regarding the role of this position. However, a more conserved fragment comprising nearby residues 579–613 of the loop (which includes the 602 residue) and another fragment containing the 619 amino acid, interact with and perturb cellular and model membranes [28–30]. It has been hypothesized that the loop may bind to and destabilize the host cell membrane, as well as stabilize the trimeric helical hairpin, then favoring the formation of the fusion pore [28]. Thus, a hydrophobic 619 residue in R5X4 strains may enhance the interaction of the loop with membranes. On the other hand, since the loop is part of a wide region composing the gp120-gp41 interface [27, 31], it may influence the efficiency of gp120 shedding. It has been demonstrated that gp120 shedding requires the presence of CXCR4 [5], although a similar analysis for CCR5 is still lacking.

HR2 amino acids 636, 640, and 641 may participate in coreceptor recognition by interacting with the gp120 coreceptor binding site. The HR2-based peptide T-20 interacts with peptides derived from the bridging sheet [32], and can block the interaction of gp120-CD4 complexes with the CXCR4 coreceptor through binding a region near the base of the gp120 V3 loop [33]. Recently, Moseri and cols. showed that T-20 binds to the conserved region 4 of R5 gp120 trough mostly hydrophobic interactions [34]. On the other hand, the direct interaction of the gp41 ectodomain with the coreceptor molecule has been suggested by the observation that T-20 and the related T22 peptide, inhibited the binding to CXCR4 of the anti-CXCR4 HIV-blocking antibody 12G5 [35]. CXCR4, but not CCR5, contains a highly hydrophobic groove in the region located between the second and third extracellular loops. Since the second extracellular loop is critical for coreceptor function [36, 37], this region represents a putative site for interaction with the hydrophobic residues of the gp41 ectodomain of R5X4 viruses. Finally, it is possible that residues 619, 640 and 641 of R5X4 gp41 proteins strengthen the interaction of this molecule with membrane lipids. HR1 and HR2 peptides interact with membrane vesicles and it has been proposed that they play an important role in the interaction of gp41 with the viral and cellular membranes during the opening of the fusion pore [38–42]. Current structural models indicate that residues 636, 640, 641 are not part of the HR1-HR2 interface in the six-helix bundle [43], so they would be exposed on this structure and available for membrane interactions in late stages of the fusion process, contributing to fusogenicity and pathogenicity of R5X4 viruses (Fig. 4).

Importantly, correlation analysis revealed that the hydropathy index of pairs 602–640, 602–723, 619–640, 636–640, 640–662, and 640–756, covariate with higher correlation coefficients in the R5X4 group than in the R5 and X4 groups (Table 4), suggesting a complementary functionality of these residues for determination of the R5X4 phenotype. The positive covariation of the 619–640 and 640–756 pairs suggests a joint hydrophobic effect of these positions in R5X4 viruses for membrane lipid interactions (Fig. 2). On the other hand, the negative covariation observed for positions with opposed hydropathy tendencies (602–640, 602–723, 636–640 and 640–662) remarks the importance of the concurrence of hydrophilicity at positions 602, 636 and 662 (Fig. 2) for the R5X4 phenotype. In particular, the participation of position 640 in five of six covariations and the exposed position of this residue on the six-helix bundle structure (Fig. 4), suggest an important role of this residue for the R5X4 phenotype.

Residue 723 is part of a region in the C-terminal tail that may be transiently exposed on the surface virus and infected cells and is so called the minor ectodomain [23–25], while position 756 locates in a region that may constitute a third membrane spanning domain (MSD3) during exposition of the minor ectodomain [25, 26]. A hydrophobic residue at this position may favor the exposure of the minor ectodomain, although with still unsuspected consequences.

A less restrictive analysis (Q_FD_ = 0.1) rendered additional positions located at different domains of gp41 and again, only for the R5X4 vs. R5 comparison. Thus, statistical analysis suggests a role for gp41 in the R5X4 virus phenotype.

Our analysis of the relationship of the gp41 sequence with virus phenotype did not yield differences between the X4 and R5 groups. It is well known that V3 gp120 residues influence the macrophage-tropic R5 (M-R5) and T-cell tropic (T-X4) viral phenotypes [44, 45], yet the role of V3 as a major determinant of phenotype is less clear in the case of dually tropic viruses [8]. Since our analysis was performed independently of the gp120 sequence, it is likely that we only observed residues influencing the R5-R5X4 shift in gp41, whereas residues in gp120 would be significant in determination of the R5 and X4 phenotypes.

## Conclusions

R5 and R5X4 are the two main classes of viruses found in the circulation of patients with HIV-1 infection. Our analysis suggests that physicochemical properties of the variable amino acid residues at positions 602, 619, 636, 640, 641, 662, 723 and 756 of gp41 may contribute to enhanced virus-host membrane fusion of R5X4 viruses respective to R5 viruses.

## Methods

### HIV-1 sequences

A total of 2823 gp41 amino acid sequences from all main subtypes with defined coreceptor usage available in Los Alamos HIV database (19) were downloaded as follows: 2346 R5, 197 X4 and 280 R5X4. Consensus were constructed for homologous sequences (i.e. those derived from the same patient and having the same tropism), by using the Consensus Maker software available in Los Alamos HIV database website (19). As a result, a set of 773 sequences was obtained and classified according to coreceptor usage: 621 R5, 73 X4, and 79 R5X4. Table 1 presents the relative abundance of consensus sequences from strains with a given coreceptor tropism in the main genetic subtypes. Sequences from B and C clades were the most abundant and belonged mainly to the R5 group (81.4 and 87.2 %, respectively). Recombinant subtypes were grouped together in “others”. Sequences were aligned with respect to the reference HXBc2 strain by using the Clustal W subroutine of the MEGA 5.2 software.

### Entropy determination

The softwares Entropy-one and Entropy-two available from the Los Alamos HIV database were employed to localize non-conserved regions of gp41 by evaluating Shannon’s entropy (*S*_*k*_) for each aligned position:

$$ {S}_k={\displaystyle \sum_r\;f\left(r,k\right)\kern0.1em { \log}_2}\;f\left(r,k\right) $$

where *f(r, k)* is the frequency of the residue *r* at position *k*. Entropy differences between groups at site *k* were calculated as S_kB_-S_kA_, where *A* and *B* designate either R5, X4, or R5X4 virus sequences. The entropy per site *S*_*k*_ and the mean entropy S_M_ for a set of sequences satisfy the relation

$$ {S}_M=\frac{1}{N}\sum_{k=1}^N{S}_k $$

where N is the total number of sites considered in the analysis.

### Statistical analysis

Independence of the HI or Q distributions at a given amino acid position between coreceptor groups was determined by the Mann–Whitney *U* test. On the other hand, the hypothesis of linkage of coreceptor usage with the hydrophobic/hydrophilic or charged/uncharged character of residues was tested by means of a *χ*2 analysis. Correction for multiple tests was performed by means of Benjamini-Hochberg procedure [46] by considering either false discovery rates Q_FD_ = 0.05 and Q_FD_ = 0.10.

### Correlation analysis

A covariance analysis was performed on HI and Q values for pairs of statistically significant positions. Covariation was expressed in terms of Pearson’s correlation coefficient *r*.

## Additional files

Additional file 1: Table S1. Variability (entropy) and statistical correlation between coreceptor usage and hydropathy index of gp41 residues. (DOCX 30 kb) Additional file 2: Table S2. Statistical correlation between coreceptor usage and charge of gp41 residues (DOCX 19 kb) Additional file 3: Table S3. Pearson’s correlation coefficients (*r*) for charge in gp41 alignments of R5, X4, and R5X4 sequences (DOCX 14 kb) Additional file 4: Figure S1. Gp41 coordinates - Homology model (PDB 598 kb)

## References

[1] Schuitemaker H, Van’t Wout AB, Lusso P (2011). Clinical significance of HIV-1 coreceptor usage. J Transl Med.

[2] Steckbeck JD, Craigo JK, Barnes CO, Montelaro RC (2011). Highly conserved structural properties of the C-terminal tail of HIV-1 gp41 protein despite substantial sequence variation among diverse clades: implications for functions in viral replication. J Biol Chem.

[3] Rizzuto CD, Wyatt R, Hernandez-Ramos N, Sun Y, Kwong PD, Hendrickson WA (1998). A conserved HIV gp120 glycoprotein structure involved in chemokine receptor binding. Science.

[4] Blumenthal R, Durell S, Viard M (2012). HIV entry and envelope glycoprotein-mediated fusion. J Biol Chem.

[5] Chien MP, Jiang S, Chang DK (2008). The function of coreceptor as a basis for the kinetic dissection of HIV type 1 envelope protein-mediated cell fusion. FASEB J.

[6] Hartley O, Klasse PJ, Sattentau QJ, Moore JP (2005). V3: HIV’s switch-hitter. AIDS Res Hum Retroviruses.

[7] Edo-Matas D, Rachinger A, Setiawan LC, Boeser-Nunnink BD, van’t Wout AB, Lemey P (2012). The evolution of human immunodeficiency virus type-1 (HIV-1) envelope molecular properties and coreceptor use at all stages of infection in an HIV-1 donor-recipient pair. Virology.

[8] Ghaffari G, Tuttle DL, Briggs D, Burkhardt BR, Bhatt D, Andiman WA (2005). Complex determinants in human immunodeficiency virus type 1 envelope gp120 mediate CXCR4-dependent infection of macrophages. J Virol.

[9] Nabatov AA, Pollakis G, Linnemann T, Kliphius A, Chalaby MI, Paxton WA (2004). Intrapatient alterations in the human immunodeficiency virus type 1 gp120 V1V2 and V3 regions differentially modulate coreceptor usage, virus inhibition by CC/CXC chemokines, soluble CD4, and the b12 and 2G12 monoclonal antibodies. J Virol.

[10] Edo-Matas D, van Dort KA, Setiawan LC, Schuitemaker H, Kootstra NA (2011). Comparison of in vivo and in vitro evolution of CCR5 to CXCR4 coreceptor use of primary human immunodeficiency virus type 1 variants. Virology.

[11] Daumer M, Kaiser R, Klein R, Lengauer T, Thiele B, Thielen A (2011). Genotypic tropism testing by massively parallel sequencing: qualitative and quantitative analysis. BMC Med Inform Decis Mak.

[12] Seclen E, Soriano V, Gonzalez MM, Gomez S, Thielen A, Poveda E (2011). High concordance between the position-specific scoring matrix and geno2pheno algorithms for genotypic interpretation of HIV-1 tropism: V3 length as the major cause of disagreement. J Clin Microbiol.

[13] Huang W, Toma J, Fransen S, Stawiski E, Reeves JD, Whitcomb JM (2008). Coreceptor tropism can be influenced by amino acid substitutions in the gp41 transmembrane subunit of human immunodeficiency virus type 1 envelope protein. J Virol.

[14] Svicher V, Balestra E, Cento V, Sarmati L, Dori L, Vandenbroucke I (2011). HIV-1 dual/mixed tropic isolates show different genetic and phenotypic characteristics and response to maraviroc in vitro. Antiviral Res.

[15] Anastassopoulou CG, Ketas TJ, Depetris RS, Thomas AM, Klasse PJ, Moore JP (2011). Resistance of a human immunodeficiency virus type 1 isolate to a small molecule CCR5 inhibitor can involve sequence changes in both gp120 and gp41. Virology.

[16] Taylor BM, Foulke JS, Flinko R, Heredia A, DeVico A, Reitz M (2008). An alteration of human immunodeficiency virus gp41 leads to reduced CCR5 dependence and CD4 independence. J Virol.

[17] Sanders RW, Korber B, Lu M, Berkhout B, Moore JP. Mutational Analyses and Natural Variability of the gp41 Ectodomain. In: Publishing D, editor. HIV Molecular Immunology 2002. Los Alamos National Laboratory, New Mexico: DIANE Publishing; 2002.

[18] Diez-Fuertes F, Delgado E, Vega Y, Fernandez-Garcia A, Cuevas MT, Pinilla M (2013). Improvement of HIV-1 coreceptor tropism prediction by employing selected nucleotide positions of the env gene in a Bayesian network classifier. J Antimicrob Chemother.

[19] Dimonte S, Mercurio F, Svicher V, D’Arrigo R, Perno C-F, Ceccherini-Silberstein F (2011). Selected amino acid mutations in HIV-1 B subtype gp41 are Associated with Specific gp120(V3)signatures in the regulation of Co-Receptor usage. Retrovirology.

[20] Thielen A, Lengauer T, Swenson LC, Dong WW, McGovern RA, Lewis M (2011). Mutations in gp41 are correlated with coreceptor tropism but do not improve prediction methods substantially. Antivir Ther.

[21] Aiamkitsumrit B, Dampier W, Antell G, Rivera N, Martin-Garcia J, Pirrone V (2014). Bioinformatic analysis of HIV-1 entry and pathogenesis. Curr HIV Res.

[22] Jiang X, Feyertag F, Meehan C, McCormack G, Travers SA, Craig C et al. Characterising the diverse mutational pathways associated with R5-tropic maraviroc resistance: HIV-1 that uses the drug-bound CCR5 coreceptor. J Virol. 2015;89(22):11457-72. 10.1128/JVI.01384-15 PMC4645647 26339063

[23] Chanh TC, Dreesman GR, Kanda P, Linette GP, Sparrow JT, Ho DD (1986). Induction of anti-HIV neutralizing antibodies by synthetic peptides. EMBO J.

[24] Cleveland SM, McLain L, Cheung L, Jones TD, Hollier M, Dimmock NJ (2003). A region of the C-terminal tail of the gp41 envelope glycoprotein of human immunodeficiency virus type 1 contains a neutralizing epitope: evidence for its exposure on the surface of the virion. J Gen Virol.

[25] Heap CJ, Reading SA, Dimmock NJ (2005). An antibody specific for the C-terminal tail of the gp41 transmembrane protein of human immunodeficiency virus type 1 mediates post-attachment neutralization, probably through inhibition of virus-cell fusion. J Gen Virol.

[26] Hollier MJ, Dimmock NJ (2005). The C-terminal tail of the gp41 transmembrane envelope glycoprotein of HIV-1 clades A, B, C, and D may exist in two conformations: an analysis of sequence, structure, and function. Virology.

[27] York J, Nunberg JH (2004). Role of hydrophobic residues in the central ectodomain of gp41 in maintaining the association between human immunodeficiency virus type 1 envelope glycoprotein subunits gp120 and gp41. J Virol.

[28] Pascual R, Moreno MR, Villalain J (2005). A peptide pertaining to the loop segment of human immunodeficiency virus gp41 binds and interacts with model biomembranes: implications for the fusion mechanism. J Virol.

[29] Qiu J, Ashkenazi A, Liu S, Shai Y (2013). Structural and functional properties of the membranotropic HIV-1 glycoprotein gp41 loop region are modulated by its intrinsic hydrophobic core. J Biol Chem.

[30] Ashkenazi A, Faingold O, Kaushansky N, Ben-Nun A, Shai Y (2013). A highly conserved sequence associated with the HIV gp41 loop region is an immunomodulator of antigen-specific T cells in mice. Blood.

[31] Sen J, Yan T, Wang J, Rong L, Tao L, Caffrey M (2010). Alanine scanning mutagenesis of HIV-1 gp41 heptad repeat 1: insight into the gp120-gp41 interaction. Biochemistry.

[32] Liu S, Lu H, Niu J, Xu Y, Wu S, Jiang S (2005). Different from the HIV fusion inhibitor C34, the anti-HIV drug Fuzeon (T-20) inhibits HIV-1 entry by targeting multiple sites in gp41 and gp120. J Biol Chem.

[33] Yuan W, Craig S, Si Z, Farzan M, Sodroski J (2004). CD4-induced T-20 binding to human immunodeficiency virus type 1 gp120 blocks interaction with the CXCR4 coreceptor. J Virol.

[34] Moseri A, Biron Z, Arshava B, Scherf T, Naider F, Anglister J. The C4 region as a target for HIV entry inhibitors – NMR Mapping of the interacting segments of T20 and gp120. FEBS Journal. 2015;282(24):4643–57. 10.1111/febs.13541 PMC4715528 26432362

[35] Xu Y, Zhang X, Matsuoka M, Hattori T (2000). The possible involvement of CXCR4 in the inhibition of HIV-1 infection mediated by DP178/gp41. FEBS Lett.

[36] Wu L, LaRosa G, Kassam N, Gordon CJ, Heath H, Ruffing N (1997). Interaction of chemokine receptor CCR5 with its ligands: multiple domains for HIV-1 gp120 binding and a single domain for chemokine binding. J Exp Med.

[37] Picard L, Simmons G, Power CA, Meyer A, Weiss RA, Clapham PR (1997). Multiple extracellular domains of CCR-5 contribute to human immunodeficiency virus type 1 entry and fusion. J Virol.

[38] Kliger Y, Gallo SA, Peisajovich SG, Munoz-Barroso I, Avkin S, Blumenthal R (2001). Mode of action of an antiviral peptide from HIV-1. Inhibition at a post-lipid mixing stage. J Biol Chem.

[39] Kliger Y, Shai Y (2000). Inhibition of HIV-1 entry before gp41 folds into its fusion-active conformation. J Mol Biol.

[40] Roche J, Louis JM, Grishaev A, Ying J, Bax A (2014). Dissociation of the trimeric gp41 ectodomain at the lipid–water interface suggests an active role in HIV-1 Env-mediated membrane fusion. Proc Natl Acad Sci.

[41] Cai L, Gochin M, Liu K (2011). Biochemistry and biophysics of HIV-1 gp41 - membrane interactions and implications for HIV-1 envelope protein mediated viral-cell fusion and fusion inhibitor design. Curr Top Med Chem.

[42] Kliger Y, Peisajovich SG, Blumenthal R, Shai Y (2000). Membrane-induced conformational change during the activation of HIV-1 gp41. J Mol Biol.

[43] Chan DC, Fass D, Berger JM, Kim PS (1997). Core structure of gp41 from the HIV envelope glycoprotein. Cell.

[44] Chesebro B, Wehrly K, Nishio J, Perryman S (1996). Mapping of independent V3 envelope determinants of human immunodeficiency virus type 1 macrophage tropism and syncytium formation in lymphocytes. J Virol.

[45] Shioda T, Levy JA, Cheng-Mayer C (1991). Macrophage and T cell-line tropisms of HIV-1 are determined by specific regions of the envelope gp120 gene. Nature.

[46] Benjamini Y, Hochberg Y (1995). Controlling the False Discovery Rate: A Practical and Powerful Approach to Multiple Testing. J R Stat Soc Series B Methodol.

[47] Buzon V, Natrajan G, Schibli D, Campelo F, Kozlov MM, Weissenhorn W (2010). Crystal structure of HIV-1 gp41 including both fusion peptide and membrane proximal external regions. PLoS Pathog.

[48] Caffrey M, Cai M, Kaufman J, Stahl SJ, Wingfield PT, Covell DG (1998). Three-dimensional solution structure of the 44 kDa ectodomain of SIV gp41. EMBO J.

[49] 49. Jacobson MP, Pincus DL, Rapp CS, Day TJF, Honig B, Shaw DE, Friesner RA. A hierarchical approach to all-atom protein loop prediction. Proteins: Struct Funct Bioinf. 2004;55(2):351-67. 10.1002/prot.10613 15048827

