# Supplementary material for: Statistical correlation of nonconservative substitutions of HIV gp41 variable amino acid residues with the R5X4 HIV-1 phenotype
Source: Virol J. 2016 Feb 16;13:28. doi: 10.1186/s12985-016-0486-6 (PMC4754869; doi:10.1186/s12985-016-0486-6)
Supplement: Additional file 1: Table S1. — Variability (entropy) and statistical correlation between coreceptor usage and hydropathy index of gp41 residues. (DOCX 30 kb) [file 12985_2016_486_MOESM1_ESM.docx]

| **Supplementary table 1.** Variability (entropy) and statistical correlation between coreceptor usage and hydropathy index of gp41 residues.^a^ | | | | | | | | | | | | | | | | | | |
| --- | --- | --- | --- | --- | --- | --- | --- | --- | --- | --- | --- | --- | --- | --- | --- | --- | --- | --- |
|  |  |  |  |  |  |  |  |  |  |  |  |  |  |  |  |  |  |  |
|  |  | **Entropy (S)** | | | | **Mean hydropathy index** *(SD)* | | | | | | | **Mann-Whitney U test** | | | ***x^2^* (hydrophobic vs. hydrophilic)** | | |
| Position^b^ | Residue | **Total** | R5 | X4 | R5X4 | R5 | | X4 | | R5X4 | | | R5 vs. X4 | R5 vs. R5X4 | X4 vs. R5X4 | R5 vs. X4 | R5 vs. R5X4 | X4 vs. R5X4 |
|  |  |  |  |  |  |  |  |  |  |  | |  |  |  |  |  |  |  |
| 515 | I | 1.098 | 1.096 | 0.85 | 1.178 | 4.02 | *0.72* | 4.23 | *0.45* | 3.97 | | *0.9* | 0.019133 | 0.373510 | 0.029381 | 0.625307 | 0.014842 | 0.171161 |
| 517 | A | 0.178 | 0.089 | 0.315 | 0.509 | 1.81 | *0.28* | 1.89 | *0.7* | 2.01 | | *0.79* | 0.305881 | 0.057583 | 0.230071 | 0.343757 | 0.384665 | 0.955151 |
| 518 | L | 1.164 | 1.133 | 1.236 | 1.239 | 3.17 | *1.09* | 3.37 | *1.01* | 3.13 | | *1.07* | 0.179365 | 0.225790 | 0.089084 | na | na | na |
| **535** | M | 1.224 | 1.203 | 1.184 | 1.322 | 3.94 | *0.95* | 3.98 | *0.87* | 3.73 | | *1.06* | 0.485749 | **0.006067** | 0.028135 | 0.627268 | 0.226484 | 0.334819 |
| 583 | V | 0.935 | 0.971 | 0.772 | 0.701 | 4.18 | *0.38* | 4.21 | *0.18* | 4.14 | | *0.39* | 0.318189 | 0.107855 | 0.289061 | na | na | na |
| 588 | K | 1.032 | 0.983 | 1.199 | 1.037 | -3.92 | *0.64* | -3.66 | *1.16* | -3.84 | | *0.98* | 0.062719 | 0.404027 | 0.170211 | 0.031043 | 0.041703 | 0.936182 |
| **602** | L | 0.905 | 0.824 | 1.018 | 1.216 | 3.37 | *1.85* | 2.92 | *2.42* | 2.39 | | *2.96* | 0.200785 | 0.053075 | 0.273752 | 0.045183 | **0.000127** | 0.305390 |
| 607 | A | 1.262 | 1.235 | 1.252 | 1.363 | -0.32 | *2.24* | -0.28 | *2.24* | -0.81 | | *2.21* | 0.473366 | 0.031423 | 0.078356 | 0.901665 | 0.028855 | 0.084412 |
| 612 | A | 1.421 | 1.449 | 1.133 | 1.249 | -0.27 | *1.56* | -0.06 | *1.35* | 0 | | *1.37* | 0.247456 | 0.091382 | 0.322754 | 0.208147 | 0.170385 | 0.962432 |
| **619** | L | 1.948 | 1.987 | 1.615 | 1.564 | 0.28 | *3.13* | 0.81 | *3.07* | 1.7 | | *2.9* | 0.092707 | **0.000230** | 0.053212 | 0.262891 | **0.000218** | 0.058390 |
| 620 | E | 2.041 | 2.064 | 1.837 | 1.905 | -2.6 | *1.67* | -2.77 | *1.55* | -2.62 | | *1.69* | 0.241317 | 0.411093 | 0.363565 | 0.826916 | 0.520400 | 0.540371 |
| 621 | Q | 1.685 | 1.629 | 1.765 | 1.783 | -3.08 | *1.34* | -2.96 | *1.21* | -2.69 | | *1.71* | 0.124417 | 0.067632 | 0.389135 | 0.516005 | 0.026438 | 0.066458 |
| 624 | N | 1.588 | 1.546 | 1.719 | 1.63 | -3.13 | *1.06* | -3.02 | *1.12* | -2.91 | | *1.31* | 0.306551 | 0.261664 | 0.454601 | 0.361911 | 0.913068 | 0.334819 |
| 629 | M | 1.144 | 1.158 | 1.146 | 0.97 | 2.33 | *1.58* | 2.05 | *1.79* | 2.02 | | *1.71* | 0.147260 | 0.098088 | 0.456193 | 0.138196 | 0.707617 | 0.456486 |
| **636** | N | 1.287 | 1.274 | 1.236 | 1.221 | -2.18 | *1.41* | -2.38 | *1.35* | -2.74 | | *1.24* | 0.203751 | **0.002743** | 0.073936 | 0.398958 | 0.380253 | na |
| **640** | S | 1.999 | 2.002 | 1.801 | 1.782 | -2.45 | *1.54* | -2.32 | *1.56* | -1.9 | | *1.62* | 0.295021 | **0.000638** | 0.032047 | 0.803422 | 0.637309 | 0.606917 |
| **641** | L | 1.883 | 1.901 | 1.76 | 1.634 | 0.99 | *3.2* | 0.99 | *3.33* | 2.29 | | *2.83* | 0.342372 | **0.003018** | 0.012337 | 0.729974 | **0.000194** | 0.010558 |
| 644 | S | 2.103 | 2.122 | 2.038 | 1.799 | -2.39 | *1.73* | -2.41 | *1.78* | -2.08 | | *1.66* | 0.443714 | 0.077300 | 0.111896 | 0.677456 | 0.620693 | 0.955151 |
| 648 | E | 1.365 | 1.39 | 1.198 | 1.019 | -3 | *1.89* | -3.2 | *1.45* | -3.23 | | *1.21* | 0.404584 | 0.138866 | 0.260670 | 0.338488 | 0.271414 | 0.921349 |
| 651 | N | 1.08 | 1.063 | 0.81 | 1.243 | -2.22 | *2.66* | -2.47 | *2.43* | -1.76 | | *2.97* | 0.270467 | 0.111709 | 0.084833 | 0.442279 | 0.123930 | 0.099894 |
| 655 | K | 1.07 | 1.08 | 0.963 | 0.867 | -3.66 | *1.47* | -3.58 | *1.53* | -3.73 | | *1.14* | 0.282252 | 0.463570 | 0.345859 | 0.362901 | 0.848282 | 0.621098 |
| 658 | Q | 0.972 | 0.983 | 1.053 | 0.663 | -3.31 | *1.63* | -3.11 | *2.01* | -3.41 | | *1.18* | 0.447299 | 0.016434 | 0.081912 | 0.245641 | 0.331150 | 0.116672 |
| **662** | E | 1.106 | 1.076 | 1.113 | 1.191 | -1 | *2.63* | -1.56 | *2.59* | -1.88 | | *2.46* | 0.067986 | **0.006678** | 0.253921 | 0.133958 | 0.007628 | 0.388776 |
| 665 | K | 1.08 | 1.081 | 1.111 | 0.842 | -3.13 | *1.37* | -3.26 | *1.3* | -3.59 | | *0.89* | 0.487029 | 0.026686 | 0.067531 | 0.622016 | 0.423477 | 0.296613 |
| **667** | A | 1.191 | 1.286 | 0.845 | 0.486 | 0.12 | *2.4* | 0.65 | *2.17* | 1.19 | | *1.72* | 0.051433 | **0.000329** | 0.103816 | 0.040999 | **0.000020** | 0.051678 |
| 674 | N | 1.236 | 1.211 | 1.213 | 1.282 | -2.75 | *1.3* | -2.72 | *1.32* | -2.48 | | *1.39* | 0.419875 | 0.090866 | 0.200013 | 0.953505 | 0.986706 | 0.955151 |
| 677 | N | 1.218 | 1.178 | 1.307 | 1.306 | -3.53 | *0.74* | -3.61 | *0.57* | -3.54 | | *0.8* | 0.182082 | 0.174473 | 0.486190 | 0.329367 | 0.986706 | 0.334819 |
| 720 | H | 1.294 | 1.29 | 1.362 | 1.205 | 0.02 | *3.57* | -0.32 | *3.57* | 0.46 | | *3.49* | 0.240562 | 0.131402 | 0.081099 | 0.420944 | 0.442284 | 0.238571 |
| 721 | L | 1.639 | 1.627 | 1.595 | 1.682 | 1.78 | *2.44* | 2.19 | *2.32* | 2.07 | | *2.46* | 0.097340 | 0.156383 | 0.438182 | 0.062643 | 0.135255 | 0.738982 |
| **723** | T | 1.888 | 1.869 | 1.971 | 1.783 | -1.11 | *2.55* | -0.52 | *2.74* | -0.32 | | *2.63* | 0.049814 | **0.007461** | 0.307369 | 0.538516 | 0.032241 | 0.283175 |
| 724 | P | 1.134 | 1.088 | 1.146 | 1.34 | -2.36 | *1.19* | -2.26 | *1.46* | -2.22 | | *1.72* | 0.442588 | 0.485488 | 0.449702 | 0.136969 | 0.039245 | 0.779270 |
| 740 | R | 0.951 | 0.972 | 0.915 | 0.759 | -3.76 | *0.71* | -3.78 | *0.72* | -3.79 | | *0.61* | 0.389231 | 0.486911 | 0.423465 | 0.731518 | 0.721147 | na |
| 743 | D | 0.987 | 0.975 | 0.95 | 0.912 | -2.86 | *1.4* | -2.85 | *1.4* | -2.65 | | *1.39* | 0.444068 | 0.122278 | 0.245143 | 0.793824 | 0.177630 | 0.138617 |
| 746 | I | 1.641 | 1.686 | 1.365 | 1.392 | 2.44 | *2.91* | 2.94 | *2.51* | 2.92 | | *2.78* | 0.102147 | 0.013481 | 0.222943 | 0.303232 | 0.153709 | 0.778561 |
| 750 | N | 1.545 | 1.573 | 1.315 | 1.388 | -2.03 | *1.5* | -2.12 | *1.54* | -2.28 | | *1.52* | 0.161095 | 0.050457 | 0.324834 | 0.955821 | 0.895802 | 0.955151 |
| **756** | I | 1.19 | 1.184 | 1.191 | 1.143 | 3.09 | *1.31* | 3.32 | *1.35* | 3.57 | | *1.22* | 0.076975 | **0.004030** | 0.202667 | 0.343757 | 0.535850 | 0.296613 |
| 775 | L | 1.166 | 1.202 | 0.989 | 0.929 | 3.83 | *1.21* | 4.03 | *0.71* | 4.03 | | *0.74* | 0.393745 | 0.236026 | 0.376774 | 0.111226 | 0.090000 | 0.955151 |
| 778 | V | 1.076 | 1.113 | 0.941 | 0.712 | 2.65 | *1.53* | 3.09 | *1.4* | 2.68 | | *1.17* | 0.020337 | 0.408584 | 0.028643 | 0.223021 | 0.008052 | 0.066863 |
| 779 | T | 0.978 | 0.964 | 1.024 | 0.949 | 1.12 | *1.46* | 1.23 | *1.61* | 1.15 | | *1.58* | 0.398355 | 0.462889 | 0.448242 | 0.598474 | 0.822608 | 0.814212 |
| 781 | I | 1.424 | 1.468 | 1.248 | 1.06 | 2.17 | *2.29* | 2.43 | *2.33* | 2.13 | | *2.49* | 0.100954 | 0.352030 | 0.271553 | 0.669490 | 0.200705 | 0.211768 |
| 787 | R | 0.95 | 0.984 | 0.865 | 0.648 | -4.02 | *1.07* | -4.04 | *1.09* | -4.18 | | *0.56* | 0.432574 | 0.232220 | 0.339445 | 0.870750 | 0.334127 | 0.293273 |
| 788 | R | 1.085 | 1.125 | 0.811 | 0.91 | -2.82 | *1.85* | -3.09 | *1.78* | -3.31 | | *1.71* | 0.138125 | 0.029787 | 0.283275 | 0.620119 | 0.608391 | na |
| 788a | * | 1.108 | 1.139 | 0.916 | 0.91 | -4.02 | *0.73* | -4.06 | *0.27* | -3.84 | | *0.69* | 0.320970 | 0.025345 | 0.104965 | 0.748605 | 0.765432 | na |
| 788b | * | 1.11 | 1.139 | 1.053 | 0.825 | -3.93 | *0.89* | -4.07 | *0.49* | -4.23 | | *0.45* | 0.291180 | 0.036824 | 0.145174 | 0.649494 | 0.677247 | na |
| 788c | * | 1.244 | 1.284 | 1.127 | 0.853 | -1.79 | *3.74* | -2.65 | *3.3* | -1.31 | | *4.12* | 0.160212 | 0.469709 | 0.280257 | 0.191467 | 0.405875 | 0.109829 |
| 792 | A | 1.539 | 1.565 | 1.296 | 1.478 | 1.81 | *1.77* | 1.76 | *1.6* | 1.7 | | *1.67* | 0.457838 | 0.314719 | 0.387167 | 0.576318 | 0.931416 | 0.621537 |
| 801 | Q | 1.11 | 1.143 | 0.923 | 0.89 | -1.25 | *3.32* | -1.42 | *3.28* | -1.18 | | *3.47* | 0.411159 | 0.441877 | 0.483412 | 0.645365 | 0.764129 | 0.566502 |
| 804 | S | 1.157 | 1.156 | 1.089 | 1.099 | 0.03 | *1.71* | 0.4 | *2.01* | 0.38 | | *2.01* | 0.255361 | 0.331902 | 0.439022 | 0.137037 | 0.135629 | 0.976146 |
| 805 | Q | 0.932 | 0.938 | 0.822 | 0.911 | -1.95 | *3.13* | -2.15 | *2.95* | -2.63 | | *2.65* | 0.486457 | 0.044158 | 0.097652 | 0.512634 | 0.079345 | 0.382659 |
| 809 | N | 1.446 | 1.434 | 1.522 | 1.278 | -2.25 | *2.88* | -2.15 | *2.73* | -2.03 | | *3.02* | 0.141940 | 0.043101 | 0.350140 | 0.616998 | 0.642730 | 0.469984 |
| 817 | A | 0.992 | 0.981 | 0.96 | 0.986 | 0.47 | *1.44* | 0.74 | *1.44* | 0.54 | | *1.36* | 0.071773 | 0.279935 | 0.244200 | 0.094728 | 0.403950 | 0.511916 |
| 818 | T | 1.126 | 1.133 | 1.086 | 1.01 | 1.95 | *2.54* | 1.26 | *2.43* | 1.67 | | *2.56* | 0.034307 | 0.217290 | 0.217732 | 0.057992 | 0.316053 | 0.476416 |
| 829 | V | 0.926 | 0.903 | 0.924 | 1.046 | 4.25 | *0.55* | 4.27 | *0.45* | 4.12 | | *0.75* | 0.331502 | 0.194541 | 0.371944 | 0.629636 | 0.615720 | na |
| 832 | V | 1.527 | 1.535 | 1.434 | 1.489 | 3.76 | *1.17* | 3.86 | *1.02* | 3.7 | | *1.23* | 0.339615 | 0.431504 | 0.435606 | 0.720782 | 0.910604 | 0.714898 |
| 833 | V | 1.662 | 1.68 | 1.586 | 1.471 | 3.1 | *1.66* | 3.23 | *1.41* | 3.32 | | *1.5* | 0.454362 | 0.206101 | 0.288878 | 0.070667 | 0.322624 | 0.421483 |
| 836 | A | 1.532 | 1.53 | 1.342 | 1.478 | 2.97 | *1.59* | 2.68 | *1.46* | 3 | | *1.47* | 0.055636 | 0.488775 | 0.088774 | 0.048061 | 0.417796 | 0.202130 |
| 837 | C | 1.776 | 1.754 | 1.688 | 1.805 | 1.05 | *1.78* | 0.77 | *1.89* | 0.95 | | *1.88* | 0.018880 | 0.442998 | 0.049239 | 0.344979 | 0.266218 | 0.921039 |
| **841** | R | 1.23 | 1.263 | 1.022 | 0.898 | 2.14 | *3.13* | 2.6 | *2.89* | 3.22 | | *2.09* | 0.034788 | **0.000906** | 0.200679 | 0.407217 | 0.014554 | 0.166975 |
| ^a^ Bold characters indicate positions with significant *p* values after Benjamini-Hochberg correction for multiple test.  ^b^ The residue number is based on the sequence of HXB2 gp120. | | | | | | | | | | |  |  |  |  |  |  |  |  |
