# Supplementary material for: Statistical correlation of nonconservative substitutions of HIV gp41 variable amino acid residues with the R5X4 HIV-1 phenotype
Source: Virol J. 2016 Feb 16;13:28. doi: 10.1186/s12985-016-0486-6 (PMC4754869; doi:10.1186/s12985-016-0486-6)
Supplement: Additional file 2: Table S2. — Statistical correlation between coreceptor usage and charge of gp41 residues (DOCX 19 kb) [file 12985_2016_486_MOESM2_ESM.docx]

| **Supplementary table 2**. Statistical correlation between coreceptor usage and charge of gp41 residues.^a^ | | | | | | | |
| --- | --- | --- | --- | --- | --- | --- | --- |
|  |  |  |  |  |  |  |  |
|  |  | **Mann-Whitney U test** | | | ***x^2^* (charged vs. uncharged)** | | |
| Position^b^ | Residue | R5 vs. X4 | R5 vs R5X4 | X4 vs. R5X4 | R5 vs. X4 | R5 vs R5X4 | X4 vs. R5X4 |
|  |  |  |  |  |  |  |  |
| 515 | I | 0.499913 | 0.500000 | 0.500000 | na | na | na |
| 517 | A | 0.499747 | 0.500000 | 0.500000 | na | na | na |
| 518 | L | 0.499732 | 0.500000 | 0.500000 | na | na | na |
| 535 | M | 0.500000 | 0.500000 | 0.500000 | na | na | na |
| 583 | V | 0.500000 | 0.500000 | 0.500000 | na | na | na |
| 588 | K | 0.160049 | 0.268532 | 0.376719 | 0.103533 | 0.824903 | 0.824903 |
| **602** | L | 0.249509 | 0.030805 | 0.195607 | 0.115875 | **0.000047** | 0.167596 |
| 607 | A | 0.446352 | 0.444430 | 0.500000 | 0.398958 | 0.380253 | na |
| 612 | A | 0.339905 | 0.407170 | 0.441659 | 0.382550 | 0.105583 | 0.296613 |
| 619 | L | 0.280392 | 0.421899 | 0.274213 | 0.724373 | 0.181646 | 0.178077 |
| 620 | E | 0.238424 | 0.168277 | 0.430362 | 0.642609 | 0.305761 | 0.686034 |
| 621 | Q | 0.012637 | 0.019235 | 0.433330 | 0.003603 | 0.014075 | 0.709903 |
| 624 | N | 0.172221 | 0.016246 | 0.206524 | 0.840020 | 0.085438 | 0.265990 |
| 629 | M | 0.427765 | 0.437367 | 0.490402 | 0.403680 | 0.466258 | 0.936182 |
| **636** | **N** | 0.413183 | **0.000188** | 0.002526 | na | **0.000044** | 0.003069 |
| 640 | S | 0.050335 | 0.406542 | 0.077412 | na | 0.855218 | 0.059151 |
| 641 | L | 0.374454 | 0.468457 | 0.380592 | 0.282333 | 0.143795 | 0.051325 |
| 644 | S | 0.227395 | 0.442816 | 0.234439 | 0.322821 | 0.015661 | 0.009494 |
| 648 | E | 0.256014 | 0.070616 | 0.281192 | 0.805883 | 0.376506 | 0.659340 |
| 651 | N | 0.358079 | 0.281917 | 0.442828 | 0.829046 | 0.384243 | 0.585798 |
| 655 | K | 0.470213 | 0.336131 | 0.357503 | 0.893532 | 0.177522 | 0.328225 |
| **658** | Q | 0.420180 | 0.003752 | 0.033537 | 0.716941 | **0.001047** | 0.024680 |
| 662 | E | 0.395580 | 0.278831 | 0.408297 | 0.588662 | 0.235039 | 0.644045 |
| 665 | K | 0.331556 | 0.032285 | 0.048306 | 0.923507 | 0.016395 | 0.068789 |
| 667 | A | 0.411965 | 0.207432 | 0.327633 | 0.104721 | 0.005526 | 0.248509 |
| 674 | N | 0.042035 | 0.013275 | 0.374392 | 0.031105 | 0.015514 | 0.891107 |
| 677 | N | 0.199968 | 0.325686 | 0.378059 | 0.345525 | 0.644716 | 0.709903 |
| 720 | H | 0.362300 | 0.202086 | 0.186915 | 0.676627 | 0.323609 | 0.293265 |
| 721 | L | 0.432639 | 0.429382 | 0.397202 | 0.528124 | 0.529334 | 0.350229 |
| 723 | T | 0.237551 | 0.240021 | 0.491007 | 0.197246 | 0.197602 | 0.967903 |
| 724 | P | 0.486703 | 0.438731 | 0.464830 | 0.938825 | 0.249318 | 0.470405 |
| 740 | R | 0.445492 | 0.446234 | 0.418100 | 0.783386 | 0.658867 | 0.592187 |
| 743 | D | 0.318936 | 0.167942 | 0.360990 | 0.501192 | 0.303462 | 0.814391 |
| 746 | I | 0.204927 | 0.465246 | 0.285902 | 0.388585 | 0.810027 | 0.599160 |
| 750 | N | 0.183662 | 0.442479 | 0.279724 | 0.721486 | 0.883014 | 0.867859 |
| 756 | I | 0.500000 | 0.500000 | 0.500000 | na | na | na |
| 775 | L | 0.500000 | 0.500000 | 0.500000 | na | na | na |
| 778 | V | 0.500000 | 0.500000 | 0.500000 | na | na | na |
| 779 | T | 0.455423 | 0.473909 | 0.447717 | 0.444039 | 0.677904 | 0.338141 |
| 781 | I | 0.499933 | 0.500000 | 0.500000 | na | na | na |
| 787 | R | 0.489107 | 0.341085 | 0.374220 | 0.890945 | 0.304712 | 0.342052 |
| 788 | R | 0.167274 | 0.041302 | 0.289504 | 0.265671 | 0.044146 | 0.511618 |
| 788a | * | 0.270250 | 0.348124 | 0.233519 | 0.150519 | 0.329737 | 0.055818 |
| 788b | * | 0.235242 | 0.048807 | 0.224797 | 0.456595 | 0.064398 | 0.347110 |
| 788c | * | 0.337786 | 0.445599 | 0.349048 | 0.613373 | 0.869586 | 0.624989 |
| 792 | A | 0.491113 | 0.491640 | 0.499999 | 0.731310 | 0.722630 | na |
| 801 | Q | 0.384493 | 0.475606 | 0.392378 | 0.211679 | 0.801653 | 0.171161 |
| 804 | S | 0.445818 | 0.443229 | 0.500000 | 0.398958 | 0.380253 | na |
| 805 | Q | 0.326429 | 0.233246 | 0.189403 | 0.408195 | 0.195700 | 0.125019 |
| 809 | N | 0.234291 | 0.028618 | 0.195943 | 0.214272 | 0.005030 | 0.197648 |
| 817 | A | 0.490761 | 0.491140 | 0.500000 | 0.731518 | 0.721147 | na |
| 818 | T | 0.500000 | 0.500000 | 0.500000 | na | na | na |
| 329 | V | 0.500000 | 0.500000 | 0.500000 | na | na | na |
| 832 | V | 0.490518 | 0.490839 | 0.500000 | 0.731310 | 0.720932 | na |
| 833 | V | 0.500000 | 0.500000 | 0.500000 | na | na | na |
| 836 | A | 0.425031 | 0.500000 | 0.442276 | 0.003541 | na | 0.296613 |
| 837 | C | 0.489759 | 0.490244 | 0.500000 | 0.731310 | 0.720932 | na |
| 841 | R | 0.272076 | 0.048208 | 0.221965 | 0.341554 | 0.007763 | 0.114945 |
| ^a^ Bold characters indicate positions with significant *p* values after Benjamini-Hochberg correction for multiple tests. | | | | | |  |  |
| ^b^ The residue number is based on the sequence of HXB2 gp120. | | | | | |  |  |
