# Supplementary material for: Statistical correlation of nonconservative substitutions of HIV gp41 variable amino acid residues with the R5X4 HIV-1 phenotype
Source: Virol J. 2016 Feb 16;13:28. doi: 10.1186/s12985-016-0486-6 (PMC4754869; doi:10.1186/s12985-016-0486-6)
Supplement: Additional file 3: Table S3. — Pearson’s correlation coefficients (r) for charge in gp41 alignments of R5, X4, and R5X4 sequences (DOCX 14 kb) [file 12985_2016_486_MOESM3_ESM.docx]

|  |  | R5 | | |  | X4 | |  | | | R5X4 | | |
| --- | --- | --- | --- | --- | --- | --- | --- | --- | --- | --- | --- | --- | --- |
| Position 1 | Position 2 | ***r*** | *p* |  | ***r*** | | *p* | |  | ***r*** | | *p* |  |
| 602 | 619 | -0,01 | 4,0E-01 |  | 0,05 | | *3,5E-01* | |  | 0,23 | | *1,7E-02* |  |
| 602 | 636 | -0,25 | 1,0E-10 |  | -0,22 | | *3,2E-02* | |  | -0,36 | | *6,0E-04* |  |
| 602 | 641 | 0,02 | 3,6E-01 |  | 0,04 | | *3,5E-01* | |  | 0,20 | | *4,7E-02* |  |
| 602 | 662 | 0,12 | 1,4E-03 |  | 0,18 | | *5,6E-02* | |  | 0,20 | | *3,6E-02* |  |
| 602 | 723 | -0,12 | 1,8E-03 |  | -0,14 | | *1,2E-01* | |  | -0,19 | | *4,7E-02* |  |
| 619 | 658 | 0,02 | 2,9E-01 |  | 0,13 | | *1,2E-01* | |  | 0,20 | | *4,7E-02* |  |
| 636 | 641 | 0,11 | 4,6E-03 |  | 0,24 | | *2,2E-02* | |  | -0,07 | | *2,6E-01* |  |
| 636 | 658 | 0,35 | 7,9E-19 |  | 0,36 | | *8,5E-04* | |  | 0,29 | | *5,6E-03* |  |
| 636 | 662 | 0,11 | 2,1E-03 |  | -0,04 | | *4,0E-01* | |  | 0,09 | | *2,1E-01* |  |
| 636 | 723 | 0,27 | 3,1E-11 |  | 0,17 | | *5,9E-02* | |  | 0,02 | | *4,7E-01* |  |
| 640 | 641 | -0,10 | 5,3E-03 |  | -0,34 | | *1,8E-03* | |  | -0,15 | | *8,2E-02* |  |
| 640 | 662 | -0,16 | 2,4E-05 |  | -0,17 | | *6,4E-02* | |  | -0,01 | | *4,7E-01* |  |
| 640 | 723 | 0,11 | 4,5E-03 |  | -0,05 | | *3,4E-01* | |  | -0,08 | | *2,6E-01* |  |
| 641 | 658 | 0,14 | 3,7E-04 |  | 0,34 | | *1,8E-03* | |  | 0,19 | | *5,5E-02* |  |
| 641 | 723 | 0,14 | 2,4E-04 |  | 0,16 | | *8,6E-02* | |  | 0,03 | | *4,1E-01* |  |
| 658 | 662 | -0,17 | 1,6E-05 |  | -0,06 | | *2,9E-01* | |  | -0,18 | | *5,3E-02* |  |
| 658 | 723 | 0,10 | 9,6E-03 |  | 0,23 | | *2,7E-02* | |  | 0,12 | | *1,5E-01* |  |

**Supplementary table 3**. Pearson's correlation coefficients (*r*) for charge in gp41 alignments of R5, X4, and R5X4 sequences^a^.

^a^ Only correlations with a *p*-value less than .05 for at least one coreceptor group are shown.
